# Adaptive fitness of *Sapindus emarginatus* Vahl populations towards future climatic regimes and the limiting factors of its distribution

**DOI:** 10.1038/s41598-020-60219-8

**Published:** 2020-03-02

**Authors:** Ashish Kumar Pal, Vivek Vaishnav, Baleshwar Meena, Nalini Pandey, Tikam Singh Rana

**Affiliations:** 1 Plant Diversity, Systematics and Herbarium Division, CSIR-National Botanical Research Institute, Rana Pratap Marg, Lucknow, 226001 India; 2 Plant Nutrition and Stress Physiology Laboratory, Department of Botany, University of Lucknow, Lucknow, 226007 India

**Keywords:** Ecological genetics, Genetic markers

## Abstract

*Sapindus emarginatus* Vahl (Sapindaceae) also known as ‘Indian Soap nut’ is significantly important for saponin content in its fruits. However, its current population in India is heavily fragmented due to a lack of sustainable harvesting practices. Moreover, changing climatic regimes may further limit its distribution and possibly compromise the survival of the species in nature. The aim of the present study was to: predict the future distribution range of *S. emarginatus*; identify the bioclimatic variables limiting this distribution and to evaluate its adaptive fitness and genomic resilience towards these variables. To determine future species distribution range and identify limiting bioclimatic variables, we applied two different ecological niche models (ENMs; BioClim and MaxEnt) on real occurrence data (n = 88 locations). The adaptive fitness of the species was evaluated by quantifying the genetic variability with AFLP markers and marker-environmental associations, using AFLP-associated Bayesian statistics. We found 77% overlap between the baseline (2030) and predicted (2100) species distribution ranges, which were primarily determined by maximum temperature (T_MAX_) and mean annual precipitation (MAP). The T_MAX_ and MAP contributed 43.1% and 27.1%, respectively to ENM model prediction. Furthermore, AFLP loci significantly associated with bioclimatic variables, and T_MAX_ and MAP represent the lowest proportion (6.15%), confirming to the severe response of the species genome towards these variables. Nevertheless, the very low Linkage disequilibrium (LD) in these loci (4.54%) suggests that the current sensitivity to T_MAX_ and MAP is subject to change during recombination. Moreover, a combination of high heterozygosity (0.40–0.43) and high within-population variability (91.63 ± 0.31%) confirmed high adaptive fitness to maintain reproductive success. Therefore, the current populations of *S. emarginatus* have substantial genomic resilience towards future climate change, albeit significant conservation efforts (including mass multiplication) are warranted to avoid future deleterious impacts of inbreeding depression on the fragmented populations.

## Introduction

*Sapindus emarginatus* Vahl (Sapindaceae) also known as ‘Indian ‘Soap nut’ is a tree species native to Indian sub-continent. In India, it is considered to be originated in the Western-Ghats and later extended up to the West- Central- North Indian biogeographical regions^1^. The species is of substantial importance for global trade in soap and perfumery industries due to the oil and saponin content obtained from its leaves, fruits and seeds. The global trade of saponin from all plant sources is expected to grow up to 970 million US$ in 2023, from 950 million US$ in 2017^2^. India exports approximately 63,368 kg/year of saponin extract obtained from all plant sources to different countries^3^. The exact contribution of *S. emarginatus* to the global saponin market is unclear, but probably substantial as the species is widely used traditionally at Indian homes as detergent to wash precious clothes and ornaments and as shampoo to wash hair. Due to high commercial demand, the species has been enlisted under ‘priority species of economic importance’ in the country report of state of forest genetic resource in India^4^ and the available natural patches of the species in some places have been restored through reforestation^4^. Lack of sustainable harvesting practices has been causing problems in the natural regeneration, as the fruits are harvested without leaving seeds for its natural regeneration^5,6^, which led to fragmentation of the populations from wild and now the plants are found scattered in dry and moist deciduous forests and its periphery. Further, the distribution of the plants is subjected to significant impact due to changing climatic regimes. Therefore, there is a need to evaluate the available genetic resource of *S. emarginatus* and its adaptive fitness in projected bioclimatic regimes to ensure its sustainability.

Species distribution modeling (SDM) based on fundamental ecological niche theory has become an integral tool to provide the biogeographic extent of a species distribution^7^. With the current occurrence data of a species, the ecological niche modeling (ENM) tools such as BioClim (bioclimatic analysis and prediction system) and MaxEnt (maximum entropy), have been employed to predict the suitable niches for species population and their response toward different bioclimatic variables integrating the projected climatic regimes^8,9^. However, the actual climate tolerance of long-lived species is wider than the climatic envelope they currently occupy^10^. Specifically, the forest species are practiced to plant outside of its natural distribution for commercial, breeding and improvement purposes in monoculture. It has also been suggested for covering the commercial forestry trials, botanical gardens, and biodiversity database along with the natural distribution of a tree species to measure its climatic or environmental requirements through SDM/ENM^11^. Nevertheless, these model-based predictions are not practically successful until the species population itself is not adaptively fit^12^. A species needs to be resilient enough for local adaptation to maintain its survivability facing bottleneck selection pressures. In particular, the response of a species may vary toward dynamic extrinsic factors like changing climatic conditions. On the other hand, the existence of a high level of genetic heterogeneity within a population can lead to the increased adaptive fitness and evolutionary potential in longer-term^13–16^. An adaptive fit population is tend to transfer the same genetic characteristic to the future generations^17^. Ultimately, the response of species to the predicted changing climatic condition with high genetic heterogeneity leading to the adaptive fitness is of significant importance for the sustainability of population in limited distribution^18^.

The DNA-based marker systems such as dominant markers i.e. amplified fragment length polymorphism (AFLP), inter-simple sequence repeats (ISSR), DAMD (directed amplification of minisatellite DNA) and co-dominant markers i.e. simple sequence repeats (SSR) have become an important tool to evaluate the distribution of heterogeneity and structure of the plant genetic resources. Among the dominant markers, AFLP exhibits higher reproducibility^19^, robust informativeness, higher multiplex ratio, wider genome coverage, higher heritability^20^ and fewer artifacts compared to ISSR and other markers^21^. In a previous report on *S. emarginatus*, the AFLP markers revealed comparatively higher polymorphism (P%) and polymorphic information content (PIC) values than the ISSR and DAMD markers^22^. Using ISSR markers the genetic diversity and differentiation among *S. emarginatus* populations from three biogeographical regions in India has been evaluated^22^. Assuming the population in Hardy-Weinberg Equilibrium (HWE) and without consideration of spatial and geographical factors, the study resulted into three distinct groups showing affinities towards their biogeographical regions^22^. Unlike the classical genetic algorithm, the Bayesian algorithm developed for AFLP markers^23–25^ is advantageous to estimate the genetic diversity and differentiation of populations without considering of HWE and inbreeding coefficient^26^. Moreover, it is also able to handle the biased estimates of genetic diversity measures due to unequal sample sizes.

**Figure 1:**
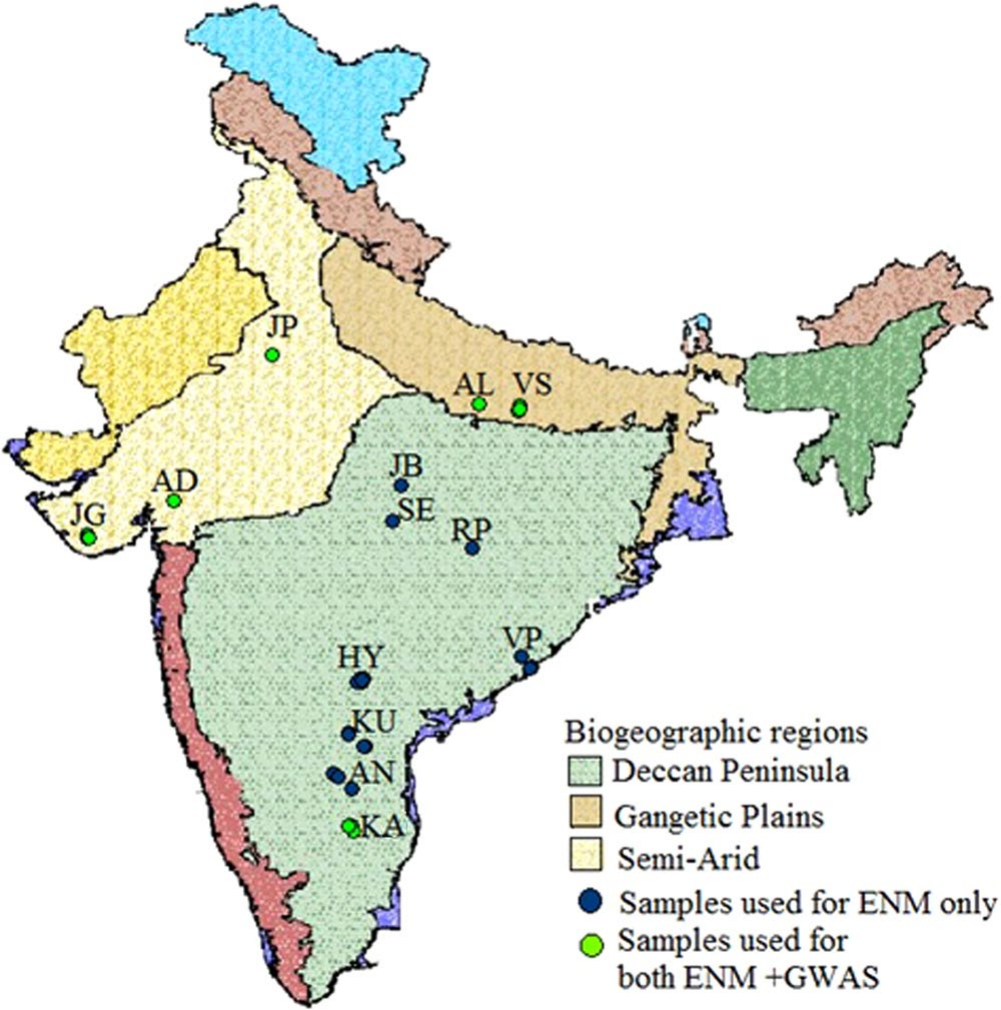
Showing *S. emarginatus* populations at different biogeographic regions sampled for the present investigation. Abbreviated location’s code is same as given in Table 4. The map was obtained from the database available in the public domain (http://wiienvis.nic.in/database/htmlpages/biozonemap.htm) and developed in the form of shapefile for modelling after georeferencing through program Q-GIS Desktop (version 3.10; http://qgis.osgeo.org).

The present investigation was performed to address the major questions: How the future climatic regime is going to affect the species population distribution? What is the state of the genomic resilience of different populations of *S. emarginatus* to respond to the projected climatic regime? What are the bioclimatic variables contributing as limiting factor in population distribution? In order to resolve these questions, ENM was performed with real occurrence data of the populations of *S. emarginatus* from three biogeographical regions in India (Fig. 1) with multi-modeling approach with two different models *viz*. BioClim and MaxEnt, comparing the predicted species distribution pattern between baseline (the year 2030) and projected (the year 2100) climatic regimes. The fitness of the populations was evaluated assessing the genetic variability using AFLP markers with Bayesian statistics. The signature of adaptation in genome of the species was evaluated using genome-wide association (GWAS) between marker and bioclimatic variables.

## Results

**Figure 2:**
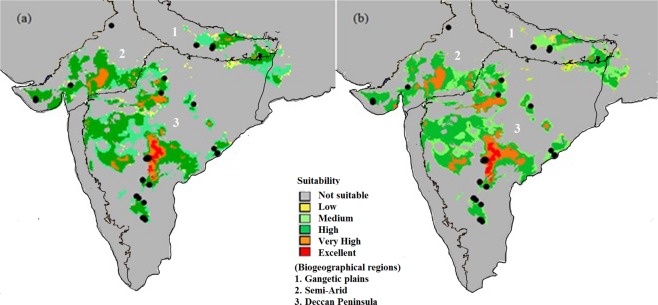
Predicted suitability of ecological niches for the occurrence of *S. emarginatus* in the years (a) 2030 and (b) 2100 in the biogeographical regions 1. Gangetic plain, 2. Semi-Arid, 3. Deccan Peninsula, resulted by MaxEnt algorithm. Black dots represent the sampled locations. The map was obtained from the shapefile of Fig. 1, and the projected output from program MaxEnt^52^ is shown with the help of program DIVA-GIS^47^.

**Figure 3:**
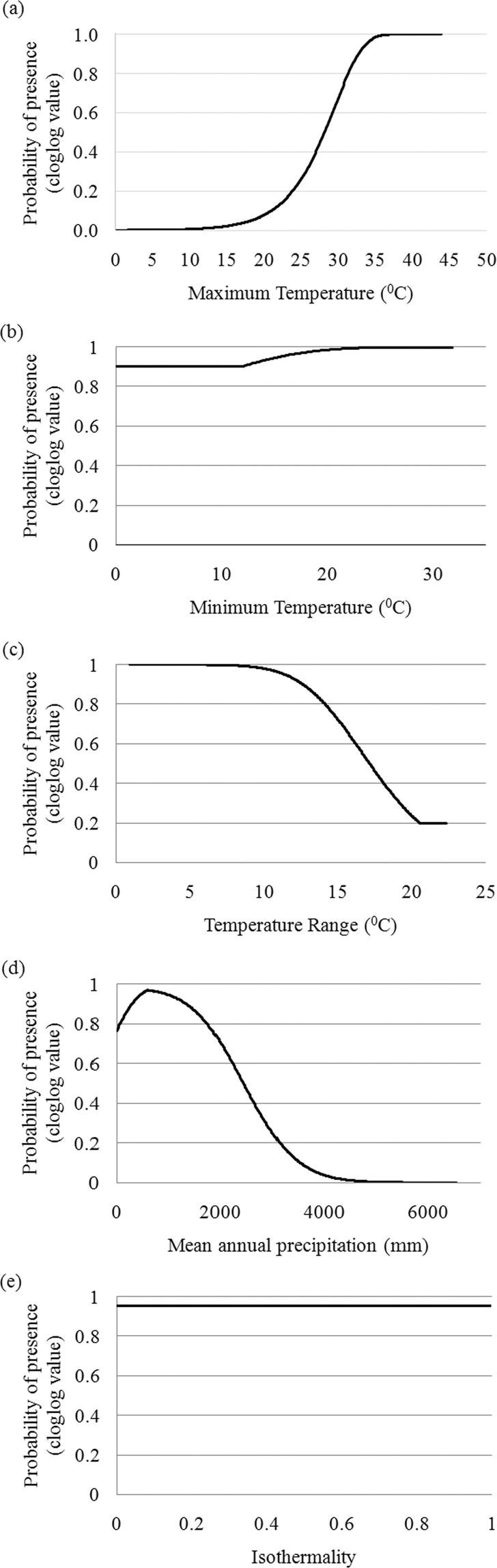
Responses of the climatic variables *viz*. (**a**) maximum temperature (°C), (**b**) minimum temperature (°C), (c) temperature range (°C), (**d**) isothermality, and (**e**) mean annual precipitation (mm) toward the probability of presence of the populations of *S. emarginatus* resulted by MaxEnt^52^.

A significant correlation (*p* < 0.05, *r* = 0.85) was found between the predictions by both the models (BioClim and MaxEnt) for the species distribution on its range of occurrence in the baseline (the year 2030) and future (the year 2100) climatic regimes. Therefore, the species distribution prediction (Fig. 2) and output obtained from the MaxEnt was applied for all further estimations. The comparison between the species distribution patterns predicted for the baseline and future climatic regimes resulted into 77% proportion of overlapping range. The niches from Deccan peninsula biogeographic region were predicted to be the most suitable for survival of the species. The niches of species occurrence at Semi-arid biogeographical region showed comparatively less suitability for the survival of species for the year 2100 (Fig. 2). During prediction, MaxEnt resulted no change in area under curve (AUC = 0.99 ± 0.0) for both climatic regimes. The prediction of limiting bioclimatic variables (by BioClim) and their responses to the probability of presence (by MaxEnt) resulted in the relative proportion of maximum temperature (T_MAX_ = 43.10%), and annual precipitation (MAP = 27.10%) with the highest contribution in the model predictions (Fig. 3, Table 1). The species niches were found with sensitive response towards T_MAX_ (<10 °C), temperature range (T_RANGE_; > 10 °C) and increased MAP (<1000 mm).

**Table 1:** Estimates of relative contribution (in %) of the climatic variables to the MaxEnt model and of AFLP loci in significant association with the climatic variables.

Approach/Models	MAP	I	T_RANGE_	T_MAX_	T_MIN_
ENM/ MaxEnt	27.10	11.30	2.20	43.10	16.40
GWAS/ MLM	7.55	20.75	39.62	7.55	24.53

ENM/MaxEnt- ecological niche modeling based on maximum entropy, GWAS/MLM – genome-wide association study based on mixed linear modeling, MAP- mean annual precipitation in mm, I- isothermality, T_RANGE_- diurnal temperature range in °C, T_MAX_- maximum temperature °C, T_MIN_- minimum temperature °C.

The AFLP primers amplified 1957 loci (103 ± 39.37 loci/primer) with 94.79 ± 6.43% polymorphism (1859 polymorphic loci), 0.65 ± 0.02 major allele frequency (MAF) and 0.33 ± 0.01 PIC (Table 2). The 49 loci amplified by the AFLP primers ACG/CTA and ACA/CAG were excluded from the analysis due to low numbers of amplified loci (<50) and further genetic analysis was performed with the 1908 loci amplified by the 17 AFLP markers (Table 2). The Bayesian model-based analysis performed with Hickory resulted into the lowest deviance information criteria (DIC) value (35357.20) supporting the suitability of full-model (inbreeding) among the four models for the data. The average heterozygosity (Hs value) generated for the locations was 0.40 ± 0.0. For the samples as a whole, panmictic heterozygosity (Ht) was 0.43, whereas θ-I and *f* (F_IS_) values were 0.18 and 0.98, respectively. The observed heterozygosity (Ho) ranged from 0.42 ± 0.0 to 0.44 ± 0.0 with an average of 0.43 ± 0.01 (Table 3) while F_ST_ value was 0.048. Allahabad (AL) population revealed the highest number of common alleles < =50%, and no rare and common (<=25%) allele was found in the populations analyzed.

**Table 2:** Genetic information revealed by the 19 AFLP primer combinations on 41 genotypes of *S. emarginatus*.

Primer Combinations EcoRI/MseI	AL	%P	PIC
AGC/CAT	99	94.95	0.32 ± 0.05
AGC/CTA	112	99.11	0.33 ± 0.03
AGC/CTG	110	100.00	0.34 ± 0.03
ACA/CAT	157	92.99	0.32 ± 0.06
ACA/CAG	27	96.30	0.33 ± 0.04
ACA/CTC	110	100.00	0.33 ± 0.03
AAC/CTG	129	100.00	0.32 ± 0.07
AAG/CAG	126	91.27	0.31 ± 0.06
ACT/CTG	110	95.45	0.33 ± 0.06
ACT/CAT	98	100.00	0.35 ± 0.03
ACT/CAA	135	100.00	0.34 ± 0.02
ACT/CAG	111	85.59	0.33 ± 0.05
ACT/CTT	156	100.00	0.34 ± 0.02
ACC/CAC	136	89.71	0.33 ± 0.06
ACC/CAT	130	90.77	0.33 ± 0.05
AGG/CAT	84	88.10	0.32 ± 0.06
AGG/CTA	53	100.00	0.34 ± 0.02
AGG/CTG	52	76.92	0.33 ± 0.04
ACG/CTA	22	100.00	0.34 ± 0.02
Average	103 ± 39.37	94.79 ± 6.43	0.33 ± 0.01

AL- the number of amplified loci, %P- the percentage of polymorphism, PIC- polymorphic information content, ±- standard deviation.

**Table 3:** Genetic diversity of *S. emarginatus* based on AFLP markers.

Regions	Location	N	CA	He	Hs	Ho
Gangetic Plain	AL	15	8	0.35 ± 0.01	0.41 ± 0	0.43 ± 0
	VS	7	2	0.30 ± 0.06	0.40 ± 0	0.43 ± 0
Semi-arid	RJ	5	0	0.28 ± 0.02	0.40 ± 0	0.44 ± 0
	GJ	9	3	0.33 ± 0.02	0.40 ± 0	0.44 ± 0
Deccan peninsula	KA	5	3	0.26 ± 0.02	0.40 ± 0	0.42 ± 0
Average (±SD)				0.30 ± 0.04	0.40 ± 0	0.43 ± 0.01

N- the number of genotypes investigated, ±- standard deviation, CA- common alleles (<=50%), He- expected heterozygosity, Hs- panmictic heterozygosity resulted from Hickory, Ho- observed heterozygosity resulted from AFLP-Surv.

**Figure 4:**
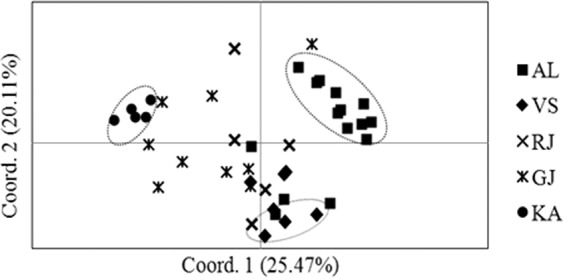
The PCoA differentiating 41 genotypes into three clusters. Few genotypes from AL, KA, and VS were found representing distinct clusters but most of them are highly admixed with the genotypes of other locations.

The Jaccard’s genetic similarity coefficient among the genotypes ranged from 0.24 to 0.61 with an average of 0.38 ± 0.05. The principal coordinate analysis (PCoA) differentiated the genotypes into three clusters (Fig. 4). Few genotypes from AL, KA, and VS populations (Table 4) were found clustering in distinct clusters but most of them are highly admixed with the genotypes of other locations. Analyzing the results in program STRUCTURE, based on the highest Delta-K value, the admixture model with independent allelic frequencies was found the most appropriate for our dataset through the program STRUCTURE HARVESTER. The most suitable cryptic population number was four (*K* = 4, Supplementary Fig. S1). The bar plot showed admixture in populations, and KA population distinctly (Supplementary Fig. S2). The populations VS, GJ, AL, and RJ were admixed, whereas GJ population was found in admixing with KA population. Populations like AL and VS were also found admixed distinctly. The F_ST_ values on these clusters ranged from 0.02 to 0.08. The pair-wise F_ST_ values among the locations ranged between 0.015 to 0.128 and the Nm exchanged among the locations ranged between 3.39 (AL-KA) to 32.39 (GJ-VS, Supplementary Fig. S2). The Mantel’s test revealed significant correlation (*p* < 0.01) between genetic distances and geographical distances matrix (Supplementary Fig. S3) and non-significant correlation (p > 0.05) was found between the number of migrants (Nm) exchanged among locations to geographic distance matrix (Supplementary Fig. S4) and altitudinal gradient (*r* = 0.59). The variation among locations was 6.46 ± 1.34% and within location among genotypes was 91.63 ± 0.31%.

**Table 4:** Geo-climatic variables at locations of *S. emarginatus* populations in different biogeographical regions of India.

Biogeographic Regions	States	Locations	N	Alt (m)	T (°C)	MAP (mm)
Gangetic Plain	Uttar Pradesh	Allahabad (AL)	15^a,b^	88 ± 0	27.80 ± 7.35	1090 ± 0
		Varanasi (VS)	7^a,b^	78.14 ± 1.34	28.14 ± 0.05	1095 ± 21.47
Semi-arid	Rajasthan	Jaipur (JP)	5^a,b^	416 ± 0	27.10 ± 0	668 ± 0
	Gujarat	Aanand (AD)	1^a,b^	105 ± 30.41	27.70 ± 0.53	1331.67 ± 74.10
		Junagarh (JG)	8^a,b^	106.25 ± 27.50	27.60 ± 0.20	1339.25 ± 55.50
Deccan peninsula	Karnataka	Kolar (KA)	5^a,b^	875 ± 34.66	25.32 ± 0.21	849.80 ± 27.13
	Telangana	Hyderabad (HY)	17^b^	547.20 ± 24.14	27.80 ± 0.2	1117 ± 33.78
	Andhra Pradesh	Vizag (VP)	10^b^	108.33 ± 91.90	28.73 ± 0.55	1189.67 ± 135.50
		Kurnool (KU)	7^b^	271 ± 41.61	29.50 ± 0.26	919 ± 30.47
		Anantpur (AN)	7^b^	424 ± 110	28.46 ± 0.68	723 ± 56.92
	Madhya Pradesh	Jabalpur (JB)	1^b^	361 ± 0	26.80 ± 0	1301 ± 0
		Seoni (SE)	1^b^	608 ± 0	26.50 ± 0	1288 ± 0
	Chhattisgarh	Raipur (RP)	4^b^	281 ± 0	28.5 ± 0	1452 ± 0
			CV%	74.40	3.90	22.51

N- the number of genotypes, Alt (m)- altitude in meters, T (°C)- the temperature in °C, MAP (mm)- mean annual precipitation in millimeter, ^a^-used for DNA profiling, ^b^-used for species distribution modeling, ± - standard deviation, CV- coefficient of variation.

**Figure 5:**
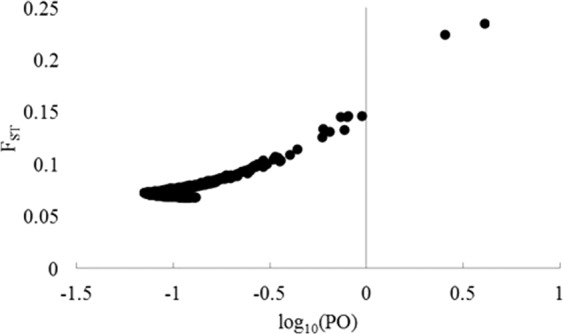
BayeScan analysis resulting F_ST_ values on Bayes Factor, i.e. Log_10_ (PO) for 1859 AFLP loci, confirms only one locus namely E-AGC/M-CTG-71 as an outlier (Log_10_ (PO) > 0.5) depicting substantial evidence to be in balancing selection.

In order to detect the outlier loci with a signature of adaptation, the only locus E-AGC/M-CTG-71 (F_ST_ = 0.23) was found with the posterior odd (log_10_PO) > 0.5 (Fig. 5). For the rest of the loci, their corresponding F_ST_ values were found real positive. Linkage disequilibrium (LD) was confirmed to avoid false-discovery in genome-wide association study (GWAS). Among all loci pairs, only 1.99% were found in significant LD (*p* < 0.01). Major LD decay was observed within a distance of 100 base pair (bp) and the length of LD block extended up to 1450 bp (Supplementary Fig. S5). The mixed linear model (MLM) based analysis detected a significant (*p* < 0.001) association of 65 loci (3.59% out of 1810 polymorphic loci) from 15 AFLP markers with bioclimatic variables and altitude (Supplementary Table S1). Among these 65 loci, the highest proportion (32.30%) was found associated with T_RANGE_ and the lowest proportion (6.15%) was with MAP and T_MAX_ (Table 1). Among 1651 possible combinations of these 65 loci, only 4.54% were found in significant (*p* < 0.001) LD.

## Discussion

The occurrence of *S. emarginatus* was found at the ecological niches exhibiting varied altitudinal ranges (CV = 74.40%), and MAP distribution (CV = 22.51%) but with a narrow temperature gradient (CV = 3.90%; Table 4). Sun *et al*. studied the natural distribution of another species of the same genus (*S. mukorossi*) which showed comparatively wider range of MAP (CV = 26.84%) and mean annual temperature (CV = 17.84%)^27^. The comparison of the CV and MAP data revealed that *S. emarginatus* might have been facing intrinsic incompetency to shift beyond the specified range of temperature gradient due to bionomic characteristics. Since the species distribution thrives on the fragmented populations in varied biogeographical regions, it may be significantly influenced by the extrinsic barriers like local conditions and selection pressures as well. Hence the adaptive fitness of the species led by the population genetic characteristics was determined to ensure the sustainability and genomic resilience of the population through dominant AFLP markers. The dominant markers have already been applied to confirm the adaptive fitness of trees^28,29^ and other plant species^30–32^. In the present study also, a high MAF (0.65 ± 0.02) of the AFLP markers and a high LD decay (1.99%) in loci-combinations supported their suitability for high-resolution GWAS^33^ confirming their frequent coverage of the species genome.

### Influence of future climatic regimes on the species population distribution

In both; BioClim and MaxEnt models, no change in AUC on projected climatic regimes confirmed a stable distribution pattern of the species population. However, our projection has confirmed the increased suitability of the niches of the species in Deccan peninsula and none of the locations of species occurrence is going to be vulnerable by the future changes, except one (JP) from the Semi-arid biogeographic region (Fig. 1), which has been found to be comparatively less suitable. This might be possible because the northern regions of India have been predicted to be more influenced by the warmer climate than other regions^34^. It can be inferred also from the results of ENM establishing the T_MAX_ as one of the limiting factors to the species distribution with the highest (43.10%) relative contribution to the model prediction (Table 1).

### The adaptive fitness of the species population

In the present investigation, a higher value (0.40–0.43) for the measures of heterozygosity (Table 3) and higher within-population variability (91.63 ± 0.31%) have been resulted. This could be possible that the species prefers xenogamy over geitonogamy due to asynchrony in flowering^35^. Moreover, the sweet incense of its flowers attracts the pollinators, like Indian honeybees^36^ that may help to admix the population through pollination between the trees scattered away from each other^35^ maintaining within population variability. The Bayesian algorithm-based analysis of genetic structure has found the admixture model with independent allele frequency as the fittest and assumed four cryptic populations for the samples. Nevertheless, a weak or negligible genetic structure (Delta K < 300, Fig. S1) confirmed high admixture of the *S. emarginatus* populations exchanging genes to limited distance. The same can be inferred from the Mantel’s test resulting no significant effect of geographical distance on a number of migrants (Nm) exchanged among the sampled genotypes (Fig. S4) leading to higher within-population variability.

The high heterozygosity of *S. emarginatus* populations in light of high admixing within-population strongly supports its adaptive fitness to maintain reproductive success. Only one locus from the AFLP markers has been found with signature of substantial selection, which confirms no possibilities of local adaptation by the populations. Nevertheless, the Bayesian model-based program HICKORY suggested the ‘full-model’ as the fittest for the data with a high value for the inbreeding coefficient (*f* = 0.98) supporting the sign of inbreeding depression on the species population. High heterozygosity with inbreeding depression in sub-divided populations represents the state of overdominance caused by heterozygote superiority^37^. The current status of the *S. emarginatus* populations in severe fragmentation along with the absence of natural regeneration through seeds, act as barrier for inter-population or long-distance out-crossing and seems to be the reason for inbreeding^38^. Inbreeding depression, causing loss of genetic variability in small or fragmented populations, may result in the reduced fitness of the offspring^39,40^. Despite inbreeding, the higher heterozygosity of the populations indicated that the population might not have crossed many generations with the inbreeding depression^41^. Therefore, being a long-lived forest tree, the reproductive success of the highly heterozygous population of *S. emarginatus* through mass multiplication leading to restoration may mask the deleterious impact of inbreeding^42^.

### Limiting factors of the species population distribution

A multi-model climate change projection for the Intergovernmental Panel on Climate Change (IPCC, Assessment Report-V) has predicted a 4% increase in warming and 14% increase in precipitation by the year 2080^43^. This increment will have some influence on the climatic envelope of fundamental ecological niches leading their shifting to suitable conditions. Temperature and rainfall have been referred to as prime contributing factors in most of the projections for future climatic regimes^44^. In our investigation also, we found that T_MAX_ and MAP with maximum contribution to the model prediction. The ENM establishes T_MAX_ and MAP as extrinsic limiting factors of the species population distribution. Since the population distribution of the species has been observed within a narrow range of temperature gradient, it may have an influence of the predicted warm climate^45^. The GWAS approach found the most of the genomic proportion covered by the AFLP markers significantly associated with T_RANGE_ and T_MIN_. It revealed the genomic resilience of the species to survive within varied T_RANGE_ and T_MIN_. It is obvious, as the species is known to be frost hardy with occurrence in tropical deciduous forests in India^46^. The least proportion of the AFLP markers was found associated with T_MAX_ and MAP establishing them as limiting factors for the genomic resilience of the species. In MLM, 3.45% of the species genome was found in significant association with the bioclimatic variables. The least proportion (6.15%) of the loci linked with T_MAX_ and MAP has confirmed the severe response of the species genome toward these variables. Nevertheless, these associations are highly dependent on high LD to transfer to future generations. We observed only 4.54% LD among the loci-combinations found in significant association with the bioclimatic variables with high LD decay (within 100 bp). Since *S. emarginatus* maintains high heterozygosity preferring xenogamy, the current status of susceptibility of the species genome responding to T_MAX_ and MAP is subject to change during recombination. In addition, the mating system preferred by *S. emarginatus* may help to avoid the deleterious effect of inbreeding on the species population. Therefore, it can be assumed that the *S. emarginatus* populations may have substantial genomic resilience toward the bioclimatic variables.

## Conclusions

To the best of our knowledge, the present study seems to be a maiden attempt to study the adaptive fitness of *S. emarginatus* populations using molecular marker technique with SDM/ENM approach. The ENM-based predictions have supported a stable distribution pattern of the species population towards the projected future climatic regime and confirmed maximum temperature and annual precipitation as the major limiting factors. However, the AFLP markers have detected the signature of adaptation on the genome of *S. emarginatus*, which supports that the species is resilient enough to survive on the threat of rising temperature and precipitation. The Deccan peninsular biogeographic region was found more suitable for the species, therefore the niches in the region can be preferred to conserve as strict nature reserves to maintain the existing genetic variability of the species populations. In light of the threat and vulnerability of ecological niches of Semi-arid region, sincere efforts can be made for screening of resilient genotypes of the species. Further, initiatives for the conservation and mass multiplication of the species are recommended to avoid the future deleterious impact of inbreeding depression on the fragmented populations. There is a scarcity of the genomic information related to *S. emarginatus*. Therefore, the 15 markers found significantly associated with the bioclimatic variables and altitudinal gradients, which can be further employed to find out their association with the functional adaptive traits.

## Methods

### Population occurrence data and sampling

The information on occurrence of the species was obtained from the global plant database like GBIF (https://www.gbif.org/occurrence/search?taxon_key=8086757), European herbaria; KEW (https://www.kew.org/search?textsearch=sapindus) and the herbarium records available at CSIR- National Botanical Research Institute, Lucknow (LWG), Central National Herbarium, Kolkata (CAL), Botanical Survey of India (Central Circle), Allahabad (BSA), Botanical Survey of India, Hyderabad (BSID) and Forest Research Institute, Dehradun (DD). The 157 GPS coordinates obtained were subjected to spatial filtration to avoid the sampling biases and equal richness based grid sampling applied through the program DIVA-GIS v7.5^47^. Remaining GPS coordinates were selected for ground-truthing to obtain the real occurrence data of *S. emarginatus* populations. The locations were visited and finally 88 coordinates were recorded at the locations with verified abundance of the species in eight Indian states representing three biogeographical regions *viz*. Gangetic plain, Semi-arid and Deccan peninsula (Table 4, Fig. 1) for ENM. For DNA profiling leaf samples were collected from 41 genotypes of five localities (Table 4, Fig. 1) representing the above biogeographical regions.

### Bioclimatic data

The data of 35 bioclimatic variables^48^ for baseline (year 2000–2030) and projected (the year 2070–2100) regimes was obtained from the website http://www.climond.org (Climond model CSIRO- MK 3.0) and extracted for the GPS coordinates of the locations using DIVA-GIS v7.5^47^ (available at http://www.diva-gis.org/) in the resolution of 2.5 m.

### Prediction of the distribution pattern

The GPS coordinates were employed for ENM to predict the potential distribution of populations determining suitable niches for the species. For confirmation of the result both, BioClim and MaxEnt based models were applied as the assembling and comparing the results from different models can give robust projection^49,50^. The BioClim algorithm was applied using the envelope method as implemented in program DIVA-GIS v7.5^47^ for baseline and predicted bioclimatic variables. MaxEnt model was applied through program MaxEnt v3.4.1^50–52^ to evaluate the response of niches to the bioclimatic variables for baseline and predicted climatic regimes. For training with threshold-depended sensitivity model (threshold = 0.5), default parameters in program setting were preferred with 50 replications and 500 iterations doing jackknife and to measure variable importance. The results obtained for two different models for two climatic regimes were correlated and the proportion of overlapping niches was determined through program ENMtools v1.8^53^ applying relative rank statistics^54^. Based on the significant correlation among the bioclimatic variables obtained through program ENMtools v1.8, finally climatic variables *viz*. minimum temperature (T_MIN_), maximum temperature (T_MAX_), temperature range (T_RANGE_), isothermality (I), and mean annual precipitation (MAP) were tested to find out the limiting factor for the species distribution.

### DNA isolation and AFLP amplification

Genomic DNA was isolated from the collected leaf samples following a modified CTAB method^22^. The conventional AFLP protocol^19^ was used with minor modifications^55^ to develop an amplification profile for the 41 genotypes on 19 AFLP primer combinations (Table 2).

### Assessment of genetic diversity

DNA profiling of 41 genotypes with 19 combinations of AFLP primers was developed based on scoring the presence (1) and absence (0) of the bands with help of program Genemapper^®^ v4.0 (Applied Biosystems, Foster City, CA, USA). The program eliminates the band profiles with short sizer peaks (<50) to avoid the error on scoring. Further, the AFLP markers with <50 amplified loci were excluded for further analysis. The profile was analyzed applying both band-based and allele frequency-based approach^26^. The mean allele frequency (MAF), polymorphism percentage (P %) and polymorphic information content (PIC) of the markers were calculated by program POWERMARKER v3.25^56^. The genetic diversity of the sampled populations was estimated using Bayesian models. We preferred the θ-statistics (*viz*. θ-I, θ-II, θ-III and G_ST_-B) implemented in program HICKORY v1.1^24^ that allows direct estimates of genetic differentiation measure (F_ST_) from dominant markers without prior assumption of inbreeding and Hardy -Weinberg Equilibrium (HWE) within population even with small sample size. Location-wise panmictic heterozygosity (Hs) and total panmictic heterozygosity (Ht) were also estimated. All these estimations were performed with 50,000 steps of burn-in, 500,000 replicates, ‘thinning’ = 20 following the best-suited model for the data amongst all the four models (‘full’, ‘*f* = 0’, ‘θ = 0’ and ‘*f*-free’ models) implemented in the program based on their corresponding DIC (deviance information criterion) value^25^. The inbreeding coefficient (*f* = F_IS_) resulted from the best-fitted model in HICKORY was incorporated in another Bayesian program AFLP-Surv v1.0^57^ with the non-uniform prior distribution of allele frequencies at 10000 permutations to estimate the observed heterozygosity (Ho). Rare (private) and common alleles (<25%) among the populations were calculated through program GenAlEx v6.5^58^. Jaccard’s genetic similarity coefficient among the genotypes was calculated by program DARWIN v5.0^59^. A principal coordinate analysis (PCoA) was performed to cluster the genotypes in different axes applying program GenAlEx v6.5. To determine the genetic structure of the sampled population with an appropriate number of cryptic population (*K*) assigned by the genotypes, Bayesian statistics-based program STRUCTURE v2.3.1^60^ was used. The project was run applying four prior-assumption with combinations of admixture/no-admixture models with correlated/independent allele frequencies on 100000 burn-in and 1000000 MCMC repeats with three-run of K from 1 to 9 for 2 ≤ K ≤ 8. The most suitable model and the best-suited ‘K’ for the data set were determined based on the highest Delta-K value resulted from an online program STRUCTURE HARVESTER^61^. The ancestry coefficient (Q) values of the genotypes were further used for mixed linear modeling (MLM) based association mapping. Pair-wise F_ST_ among populations were also calculated by program AFLP-Surv. The gene flow among populations was determined by calculating the number of migrants (Nm) among the populations^62^. To determine the effect of geographical distance and altitude on gene flow among locations, the Mantel’s tests were performed among pair-wise Nm, pair-wise geographical distance and pair-wise Euclidean distance based on altitude through program GenAlEx. Analysis of molecular variance (AMOVA) was also calculated.

### Detection of signature of local adaptation

To detect the signature of local adaptation on AFLP loci, program BAYESCAN v2.1^63^ was applied that identifies outlier loci with a Bayesian test based on a logistic regression model that decomposes F_ST_ values into a locus-specific component (α, selection effect) and a population-specific component (β, demographic effect). Twenty pilot runs of 5,000 iterations were used to estimate the distribution of α-parameters, followed by 50,000 iterations for sampling. Outlier loci with substantial signature of adaptation were identified using a 5% false discovery rate with posterior odds of >10 (log10 (PO) > 0.5). Linkage disequilibrium among loci-combinations was performed by program TASSEL v3.0^64^ to confirm the influence of demographic variables on the population genetic structure inferring LD decay. A genome-wide association study (GWAS) was performed between climatic variables (i.e. T_MIN_, T_MAX,_ T_RANGE_, I, and MAP), altitude and AFLP marker profile by program TASSEL v3.0 applying MLM to control the false-discovery due to the structured population^65,66^. The MLM considers the markers applied to the study as a fixed–effect factor and the population structure information of the sampled genotypes are considered as random effect factors. The required kinship (K) information was obtained from program TASSEL and the ancestry coefficient (Q) value was obtained from the program STRUCTURE output to implement the MLM + K + Q method. The loci significantly (p ≤ 0.01) associated with one or more climatic variables were taken into consideration. To find out the limiting factor, the proportions of AFLP markers linked with these climatic variables and altitude were calculated based on their number of loci in significant association with the climatic variables.

## Supplementary information

Appendix S1.

## Data Availability

The datasets generated and analyzed during the present study will be available from the corresponding author on reasonable request.
