# Supplementary material for: Adaptive fitness of Sapindus emarginatus Vahl populations towards future climatic regimes and the limiting factors of its distribution
Source: Sci Rep. 2020 Mar 2;10:3803. doi: 10.1038/s41598-020-60219-8 (PMC7052160; doi:10.1038/s41598-020-60219-8)
Supplement: Supplementary file 1 — Appendix S1. [file 41598_2020_60219_MOESM1_ESM.docx]

**Adaptive fitness of *Sapindus emarginatus* Vahl populations towards future climatic regimes and the limiting factors of its distribution**

Ashish Kumar Pal^1,2^, Vivek Vaishnav^1^, Baleshwar Meena^1^, Nalini Pandey^2^, Tikam Singh Rana^1,*^

^1^Plant Diversity, Systematic and Herbarium Division, CSIR-National Botanical Research Institute, Rana Pratap Marg, Lucknow -226001, India

^2^Plant Nutrition and Stress Physiology Laboratory, Department of Botany, University of Lucknow, Lucknow-226007, India

*Corresponding author e-mail: [ranatikam@gmail.com](mailto:ranatikam@gmail.com)

**Appendix S1.** Genetic structure, isolation by distance, linkage disequilibrium and marker-climatic variables association.

**/Fig.S1/ Fig.S2/ Fig.S3/ Fig.S4/ Fig.S5/Table S1**

**Fig. S1** STRUCTURE suggested the model ‘admixture with independent allele frequency (Ad_Ind) as the most fit among other models *viz.* admixture with correlated allele frequencies (Ad_Cor), no-admixture with correlated (Noad_Cor) and independent allele frequencies (Noad_Ind) and K=4 as the most suitable number of cryptic population


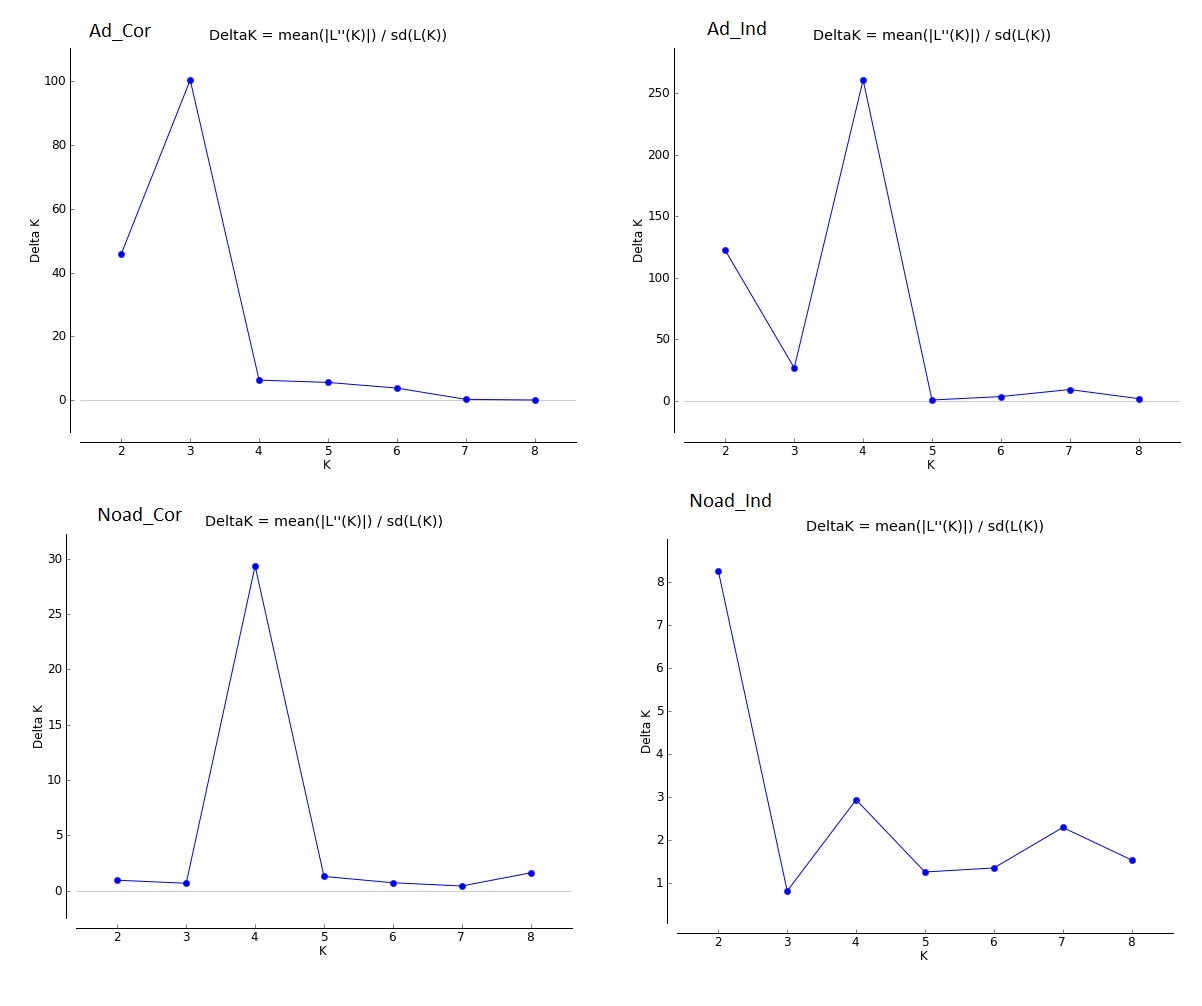


**Fig. S2** Genetic admixing among the sampled locations for ‘K=4’ resulted by STRUCTURE. The values with bidirectional arrows indicate a number of migrants (Nm) exchanged between the sampled locations. The location code is similar as given in Table 1.


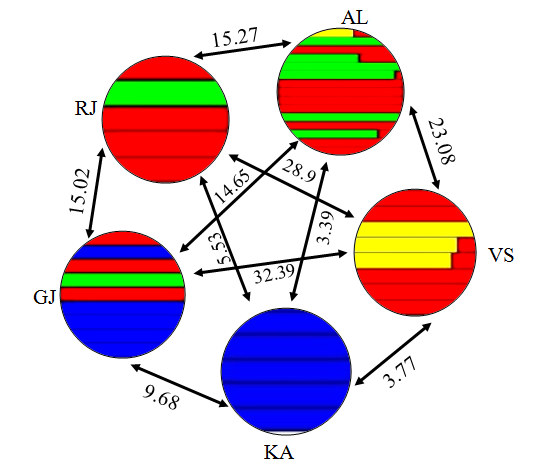


**Fig. S3** Mantel’s test between genetic distances (GD) and spatial distances among genotypes resulted in significant correlation (p<0.01)


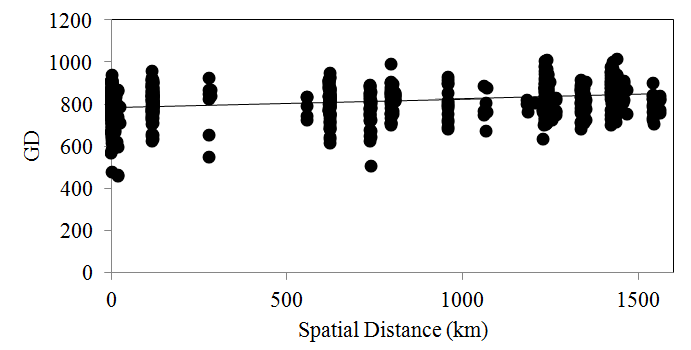


**Fig. S4** Mantel’s test between Nm exchanged between sampled locations and spatial distances between them resulted in nonsignificant correlation (p>0.1)


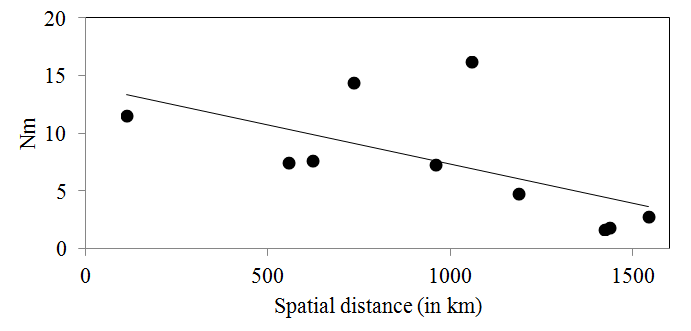


**Fig. S5** AFLP marker loci in significant LD with respective R^2^ values depicts the state of LD decay


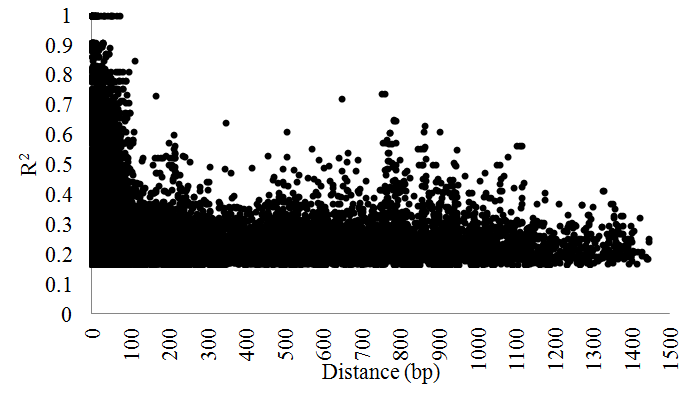


**Table S1** Number of AFLP markers loci significantly (p<0.001) associated with climatic variable and altitude resulted in GWAS/MLM based analysis

| AFLP primer  combinations  EcoRI/MseI | AFLP loci | MAP | I | T_RANGE_ | T_MAX_ | T_MIN_ | Alt | Total |
| --- | --- | --- | --- | --- | --- | --- | --- | --- |
| AAC/CTG | AAC/CTG-101 | + |  |  |  | + | + | 3 |
|  | AAC/CTG-111 |  |  | + |  |  |  | 1 |
|  | AAC/CTG-120 |  |  | + |  |  |  | 1 |
|  | AAC/CTG-18 |  |  | + |  |  |  | 1 |
|  | AAC/CTG-22 |  |  | + |  |  |  | 1 |
|  | AAC/CTG-26 |  |  |  | + |  |  | 1 |
|  | AAC/CTG-32 |  | + | + |  |  |  | 2 |
|  | AAC/CTG-56 |  |  | + |  |  |  | 1 |
|  | AAC/CTG-61 |  |  | + |  |  |  | 1 |
|  | AAC/CTG-73 |  |  |  | + |  |  | 1 |
|  | AAC/CTG-93 |  | + |  |  |  |  | 1 |
|  | AAC/CTG-94 |  |  | + |  |  |  | 1 |
| ACA/CAT | ACA/CAT-67 |  |  |  |  |  | + | 1 |
|  | ACA/CAT-121 |  |  |  |  |  | + | 1 |
| AAG/CAG | AAG/CAG49 |  |  | + |  |  |  | 1 |
|  | AAG/CAG89 |  |  |  |  | + |  | 1 |
| ACA/CAG | ACA/CAG12 |  |  |  |  | + |  | 1 |
| ACA/CTC | ACA/CTC30 |  | + | + |  |  |  | 2 |
|  | ACA/CTC39 |  |  | + |  |  |  | 1 |
|  | ACA/CTC46 |  |  | + |  |  |  | 1 |
|  | ACA/CTC47 |  |  | + |  |  |  | 1 |
|  | ACA/CTC57 |  |  | + |  |  |  | 1 |
|  | ACA/CTC63 |  | + |  |  |  |  | 1 |
|  | ACA/CTC71 |  | + | + |  |  |  | 2 |
|  | ACA/CTC80 |  |  | + |  |  |  | 1 |
|  | ACA/CTC81 |  | + | + |  |  |  | 2 |
|  | ACA/CTC86 |  |  |  |  | + |  | 1 |
|  | ACA/CTC87 |  |  |  |  | + |  | 1 |
| ACT/CAA | ACT/CAA120 |  |  |  |  | + |  | 1 |
|  | ACT/CAA121 |  |  |  |  | + |  | 1 |
|  | ACT/CAA16 |  |  |  | + |  |  | 1 |
| ACT/CAT | ACT/CAT14 |  |  | + |  |  |  | 1 |
| ACT/CTG | ACT/CTG109 |  | + |  |  |  | + | 2 |
|  | ACT/CTG110 |  |  |  |  | + | + | 2 |
|  | ACT/CTG53 |  | + |  |  | + | + | 3 |
|  | ACT/CTG9 |  | + |  |  |  |  | 1 |
|  | ACT/CTG92 |  |  | + |  |  |  | 1 |
|  | ACT/CTG93 |  | + | + |  |  |  | 2 |
| ACT/CTT | ACT/CTT49 |  |  |  |  | + |  | 1 |
|  | ACT/CTT64 |  |  |  |  | + |  | 1 |
|  | ACT/CTT87 | + |  |  |  |  |  | 1 |
|  | ACT/CTT93 |  |  |  |  |  | + | 1 |
| AGC/CAT | AGC/CAT91 |  | + |  |  |  |  | 1 |
| AGC/CTA | AGC/CTA75 | + |  |  |  |  |  | 1 |
|  | AGC/CTA81 |  |  |  |  |  | + | 1 |
|  | AGC/CTA106 |  |  |  |  |  | + | 1 |
| AGC/CTG | AGC/CTG24 |  |  |  |  | + |  | 1 |
|  | AGC/CTG30 |  |  | + |  |  |  | 1 |
|  | AGC/CTG41 |  |  |  |  | + |  | 1 |
|  | AGC/CTG70 |  |  |  | + |  |  | 1 |
|  | AGC/CTG87 | + |  |  |  |  |  | 1 |
| AGG/CAT | AGG/CAT26 |  |  |  |  |  | + | 1 |
| AGG/CTA | AGG/CTA8 |  |  |  |  |  | + | 1 |
| AGG/CTG | AGG/CTG9 |  |  |  |  |  | + | 1 |
|  | Total | 4 | 11 | 21 | 4 | 13 | 12 | 65 |
| MAP- mean annual precipitation in mm, I- isothermality, T_RANGE_- temperature range in ^0^C, T_MAX_- maximum temperature in ^0^C, T_MIN_- minimum temperature in ^0^C, Alt- altitude (m) | | | | | | | | |
